# Delivering a Post-Partum Weight Loss Intervention via Facebook or In-Person Groups: Protocol for a Randomized Feasibility Pilot Trial

**DOI:** 10.2196/15530

**Published:** 2019-11-28

**Authors:** Molly E Waring, Brooke A Libby, Tiffany A Moore Simas, Madison L Bracken, Jessica L Bibeau, Valeria Herrera, Justin Wang, Sherry L Pagoto

**Affiliations:** 1 Department of Allied Health Sciences UConn Center for mHealth and Social Media University of Connecticut Storrs, CT United States; 2 Department of Obstetrics and Gynecology University of Massachusetts Medical School/UMass Memorial Health Care Worcester, MA United States; 3 Department of Pediatrics University of Massachusetts Medical School/UMass Memorial Health Care Worcester, MA United States; 4 Department of Psychiatry University of Massachusetts Medical School/UMass Memorial Health Care Worcester, MA United States; 5 Department of Population and Quantitative Health Sciences University of Massachusetts Medical School Worcester, MA United States; 6 Department of Psychological and Brain Sciences University of Massachusetts Amherst, MA United States; 7 Social Sciences Department Community College of Rhode Island Warwick, RI United States

**Keywords:** postpartum period, weight loss, social media, pilot study

## Abstract

**Background:**

Postpartum weight retention contributes to long-term weight gain and obesity for many women. Lifestyle interventions with numerous visits are logistically challenging for many postpartum women. Delivering a lifestyle intervention via social media may overcome logistic challenges to participation in in-person weight loss programs.

**Objective:**

The objective of this study is to conduct a randomized feasibility pilot trial of a 6-month postpartum weight loss intervention delivered via Facebook or in-person groups with 72 postpartum women with overweight or obesity.

**Methods:**

Women with overweight or obesity who are 8 weeks to 12 months postpartum (N=72) will be recruited from the Hartford, Connecticut community. Eligible participants must also own an iPhone or Android smartphone and be an active Facebook user. Participants will receive a 6-month postpartum weight loss intervention based on the Diabetes Prevention Program lifestyle intervention and adapted for postpartum women. Participants will be randomized to receive the intervention via a private Facebook group or in-person group meetings. Assessments will occur at baseline, weekly during the intervention, at 6 months (at the end of the intervention), and at 12 months. Primary feasibility outcomes are recruitment, sustained participation, contamination, retention, and feasibility of assessment procedures including measurement of costs to deliver and receive the intervention. We will describe 6- and 12-month weight loss as an exploratory outcome.

**Results:**

Recruitment began in September 2018. The first wave of the intervention began in February 2019, and the second wave of the intervention is expected to begin in fall 2019. We anticipate completing follow-up assessments in fall 2020, and results will be analyzed at that time.

**Conclusions:**

Results will inform the design of a large randomized controlled trial to assess whether delivering a postpartum weight loss intervention via Facebook is noninferior for weight loss and more cost-effective than delivering the intervention via traditional in-person groups.

**International Registered Report Identifier (IRRID):**

DERR1-10.2196/15530

## Introduction

Postpartum weight retention contributes to long-term weight gain and obesity for many women [1-3]. Although average postpartum weight retention ranges from 0.5 kg to 3 kg [2,4,5], it is variable [6,7], with as many as 50% of women retaining 5 kg or more [2,4,5]. In addition to failing to lose weight gained during pregnancy, some women gain weight in the year following childbirth [8]. Having parents who are obese, especially a mother, greatly increases a child’s risk of becoming obese [9,10]. The postpartum period provides a critical period for obesity intervention [11]. Although lifestyle interventions have shown to be modestly efficacious for postpartum weight loss in randomized controlled trials [12,13], interventions with numerous in-person sessions are logistically challenging for many postpartum women [14], adding yet another strain on women at an already stressful and demanding period of life [14-20]. Treatment models for weight loss that fit into the busy lives of new moms are needed [21].

Facebook may be an effective platform for delivering evidence-based weight loss programming to postpartum women. Facebook is currently the most popular online social network [22], used by 81% of online moms [23]. Overall, 70% of Facebook users engage daily, including 43% multiple times per day [24], for at least 50 min a day on average [25]. Many women seek support about health and parenting from their Facebook network [23,26]. Facebook can connect postpartum women seeking to lose weight with each other, even across wide geographic regions. Moms, especially first-time moms, often look to other women for advice and support [26-29].

Postpartum weight loss interventions that deliver at least some content via Facebook appear promising [30-32], and additional research is ongoing [33]. We developed a postpartum weight loss intervention based on the Diabetes Prevention Program (DPP) lifestyle intervention, tailored to needs of postpartum women and for delivery by a trained weight loss counselor via a private Facebook group [30]. Our pilot work demonstrated that (1) this approach is feasible and acceptable; (2) we were able to engage participants; and (3) on average, participants achieved meaningful weight loss (average 12-week weight loss was 4.8% [SD 4.2%]; 58% lost ≥5%) [30]. Although delivering intervention content via Facebook offers many advantages, we have no reason to believe that it will be more efficacious than a traditionally delivered intervention (ie, via in-person group sessions). Instead, we hypothesize that in a large randomized trial, delivery via Facebook will be not inferior for weight loss and more cost-effective than in-person delivery. Our previous single-arm pilot study [30] did not allow us to explore the feasibility of (1) recruitment rates under conditions of randomization into online versus in-person conditions; (2) sustained participation in the Facebook-delivered intervention after 12 weeks; (3) contamination in both conditions; and (4) the feasibility of assessment procedures, particularly the measurement of costs associated with delivering and receiving the intervention. Answers to these feasibility questions are needed before conducting a large-scale noninferiority trial (Multimedia Appendix 1).

The aim of this study is to conduct a randomized feasibility pilot trial of a 6-month postpartum weight loss intervention delivered via a private Facebook group or in-person groups with 72 postpartum women with overweight or obesity to answer these feasibility questions and finalize the design of the subsequent noninferiority trial. We will examine the feasibility of recruitment, sustained participation, contamination, retention, and assessment procedures in both conditions. We will describe weight loss as an exploratory outcome. Results will provide critical preliminary data to finalize the design of a subsequent noninferiority trial.

## Methods

### Study Design

The study is a randomized feasibility pilot trial comparing delivery of a postpartum weight loss intervention via Facebook or in-person groups among women with overweight or obesity. The trial will be conducted in 2 waves. In each wave, participants will be randomized in a 1:1 ratio to either the Facebook-delivered or in-person intervention condition. The University of Connecticut institutional review board (IRB) approved this study.

### Study Population

We will recruit 72 adult women with overweight or obesity who are 8 weeks to 12 months postpartum at the time of enrollment. To be eligible to participate in this study, women will have to meet the following inclusion criteria: aged 18 years or older; body mass index ≥25 kg/m^2^ per measured height and weight at baseline; either own a scale or be willing to be provided one if needed; comfortable reading and writing in English; own an Android or iPhone smartphone; active Facebook user (defined as daily Facebook use and posts or comments at least weekly over the past 4 weeks); clearance from their primary care provider or obstetrician/gynecologist; willing to participate in either treatment condition (Facebook or in-person); available to attend in-person meetings over the 6-month study period in Hartford, Connecticut; less than 45 min travel time to intervention meetings; and willing and able to provide informed consent.

Participants who meet the following criteria will be excluded: currently pregnant; plans to become pregnant during the study period; current participation in a clinical weight loss program (online or in-person); diagnosed with type 1 or type 2 diabetes as self-reported or reported by their health care provider; medical conditions or medications affecting weight; incapable of walking a quarter of a mile unaided without stopping; pain that prevents engagement in exercise; previous bariatric surgery; planned surgery during the study period; plans to move out of the area during the study period; high depressive symptoms or suicidal ideation (a total score of 12 or higher or positive response on question number 10 on the Edinburgh Postnatal Depression Scale [EPDS] [34]); positive screen for binge eating disorder (BED) [35]; or failure to complete baseline survey, orientation webinar, or prerandomization survey. Additionally, key study personnel; spouses, dependents, or relatives of key study personnel; University of Connecticut employees who report to key study personnel; and University of Connecticut students taught by key study personnel will be excluded from participation.

### Recruitment

We will recruit postpartum women from the Hartford, Connecticut community. We will distribute flyers in local obstetric or pediatric clinics or practices; Special Supplemental Nutrition Program for Women, Infants, and Children offices; community organizations; and community venues and events. We will also recruit by posting study advertisements on ResearchMatch, Craigslist, and online social networks, including Facebook, Instagram, and Twitter. We will identify local Facebook groups relevant to postpartum women (eg, parenting groups and general community groups such as buy/sell groups) and contact the group administrators for permission to post our recruitment messages in their groups. We will also use paid advertisements on Facebook and Instagram. We will submit announcements to be included in email distribution lists at the University of Connecticut and UConn Health. Recruitment messages will include the study team’s email address and phone number and ask interested individuals to contact the team for more information about the study. If a woman contacts the study team by email, we will reply and ask her to provide her phone number or call the team. We will attempt to contact interested individuals’ phone 3 times, with a final contact via email. Research staff will screen potential participants for eligibility via phone.

In addition to recruiting postpartum women, we will post recruitment messages online targeting pregnant women whose expected due dates put them in the eligible postpartum window (eg, “are you pregnant and due before June 2019?”). Interested individuals will be asked to complete a brief online form that includes their name, contact information, and expected due date. Approximately 2 months after their expected due date, we will contact them via email or phone about their interest in the study and, if interested, screen them for eligibility.

### Baseline Assessment

A 30- to 45-min in-person baseline assessment will include provision of informed consent, measurement of height and weight, completion of a contact information sheet, and screenings for elevated depressive symptoms and BED. Study personnel will also help participants download the MyFitnessPal app and provide instruction on how to use this app to track their diet and activity. Study personnel will provide instructions on how to locate Facebook app usage data within the Facebook app and how to locate Facebook app usage data using the battery settings (iPhone users) or help participants download and use a free app to track time on Facebook (Android users).

After the baseline visit, staff will email participants a link to complete a survey via Research Electronic Data Capture (REDCap) [36]. The survey is designed to take 30 min to complete and includes measures of demographics; reproductive history [37], including gestational weight gain during the index pregnancy [38]; previous postpartum weight loss attempts [39]; quality of life [40,41]; infant feeding [42,43]; social support for diet and physical activity changes [44]; and social media use [45]. Participants will also report their Facebook use habits, including what device(s) they used to access Facebook, what proportion of use was from their smartphone (vs other devices), and the extent to which other people used Facebook on their phone [46].

We will email participants a US $20 gift card after they have completed the baseline visit and online survey. Following the baseline assessment, research staff will fax the medical clearance form to participants’ primary care provider or obstetrician/gynecologist.

### Orientation Webinar and Prerandomization Survey

Next, participants will complete a 60-min orientation webinar. The purpose of the webinar is to educate participants about what research is, review study procedures, review the importance of participation by enrolled participants, and engage them in a discussion about the advantages and challenges of participating in each treatment condition, which previous research has suggested may help participants have more realistic expectations of study participation [47], which may lead to higher retention. Following the orientation webinar, we will email participants a short online survey that includes completion of a randomization agreement, report of app-tracked time on Facebook over the past 7 days, and report of their Facebook use habits, including what device(s) they used to access Facebook, what proportion of use was from their smartphone (vs other devices), and the extent to which other people used Facebook on their phone [46].

### Randomization

Only participants who have completed the telephone screening, baseline assessments (visit and survey), orientation webinar, and prerandomization survey and for whom medical clearance has been obtained will be randomized. Randomization will occur after completing recruitment for the wave, approximately a week before the start of the intervention. We will randomize participants in a 1:1 ratio to the Facebook and in-person conditions in randomly permuted blocks of size 4 and 6. Randomization will be stratified by weeks postpartum at enrollment (8 weeks to <6 months vs 6-12 months postpartum) and smartphone type (iPhone vs Android). We will stratify randomization by months postpartum because in the absence of intervention, weight change varies across the postpartum period [48,49]. We will stratify randomization by smartphone type to ensure balance in methods for assessing time on Facebook because available tools differed by phone type during the design phase, and app or phone operating system updates may impact measurement unequally.

### Intervention

Over 6 months (25 weeks), a trained weight loss counselor will deliver a lifestyle intervention based on the DPP lifestyle intervention [50] and adapted for the needs of postpartum women. The DPP lifestyle intervention is a gold standard lifestyle intervention with ample efficacy data for weight loss [51,52]. In addition, the DPP lifestyle intervention has been successfully translated to numerous settings and populations [53], including primary care via the internet [54] and the postpartum period [55-57]. The DPP curriculum includes behavioral strategies such as self-monitoring, stimulus control, problem solving, social support, environmental restructuring, and relapse prevention [50]. The interventions will be delivered by weight loss counselors with backgrounds in nutrition/dietetics who have completed National DPP training and training by a licensed clinical psychologist with extensive experience using the DPP in our specific intervention protocols. Weight loss counselors will be supervised by a licensed clinical psychologist with extensive experience in developing and delivering in-person and online lifestyle interventions.

The intervention goals are 5% to 10% weight loss and increasing physical activity to 150 min per week of moderate-intensity physical activity. Participants will receive calorie and physical activity goals to help them achieve a healthy weight loss of 1 to 2 pounds per week. Calorie goals will account for breastfeeding, as appropriate [58]. Intervention content, individualized calorie goals, tools for self-monitoring, and access to a Pinterest board of existing online resources will be consistent across conditions; the 2 treatment conditions differ only in delivery modality.

We include intervention content specific to the needs and challenges of postpartum women, including information about energy intake needs while breastfeeding [59]; adjusted calorie goals for women who are breastfeeding [60]; quick, easy recipe ideas with emphasis on foods that appeal to children [55]; gradually increasing physical activity goals for women who were inactive during pregnancy [61]; physical activity ideas that engage infant/children; discussion around feelings of guilt for taking time away from family to exercise; discussion of barriers common to postpartum women [14-20]; managing stress related to parenting [62]; tips for getting baby on a regular sleep schedule; and partner communication skills to help to mobilize social support and assistance [63].

We will ask all participants to use MyFitnessPal to track their energy intake, physical activity, and weight. We will provide participants instructions for downloading the MyFitnessPal app and setting up their account, including setting a passcode to allow the weight loss counselor to review their diet/activity records, and tips for general use. Staff will be available to answer questions about using the website or mobile app. The weight loss counselor will email participants in both treatment conditions feedback on data entered into MyFitnessPal weekly or every 2 weeks (corresponding to the frequency of intervention sessions in the in-person condition). Participants will be withdrawn from the intervention if they report becoming pregnant to the weight loss counselor or study staff.

#### In-Person Condition

In the in-person condition, the intervention will be delivered via weekly 90-min group meetings for the first 15 weeks and then every other week in weeks 16 to 25. Intervention materials will be provided via handouts. The weight loss counselor will facilitate discussions about weekly topics. During recruitment and baseline procedures, participants will be told that they may bring their babies to in-person intervention meetings but that older children are not allowed to attend. Participants will receive up to US $5 to reimburse them for parking or bus fare.

#### Facebook Condition

The intervention will be delivered via a private (*secret*) Facebook group. *Secret* Facebook groups are restricted such that group membership is by invitation only, membership in the group does not appear in member profiles or search results, and only members can see content posted within the group [64]. During consent and the orientation webinar, study staff will outline how participants are encouraged to engage with each other (eg, read intervention posts and respond to the weight loss counselor and other participants daily), different ways to access intervention posts (eg, view in news feed and visit the group directly), and encourage participants to adjust their settings to get notifications of new posts in the group. On the first day of the intervention, the weight loss counselor posts a reminder of the format of the intervention, including encouraging participants to post at least one update a week.

**Figure 1:**
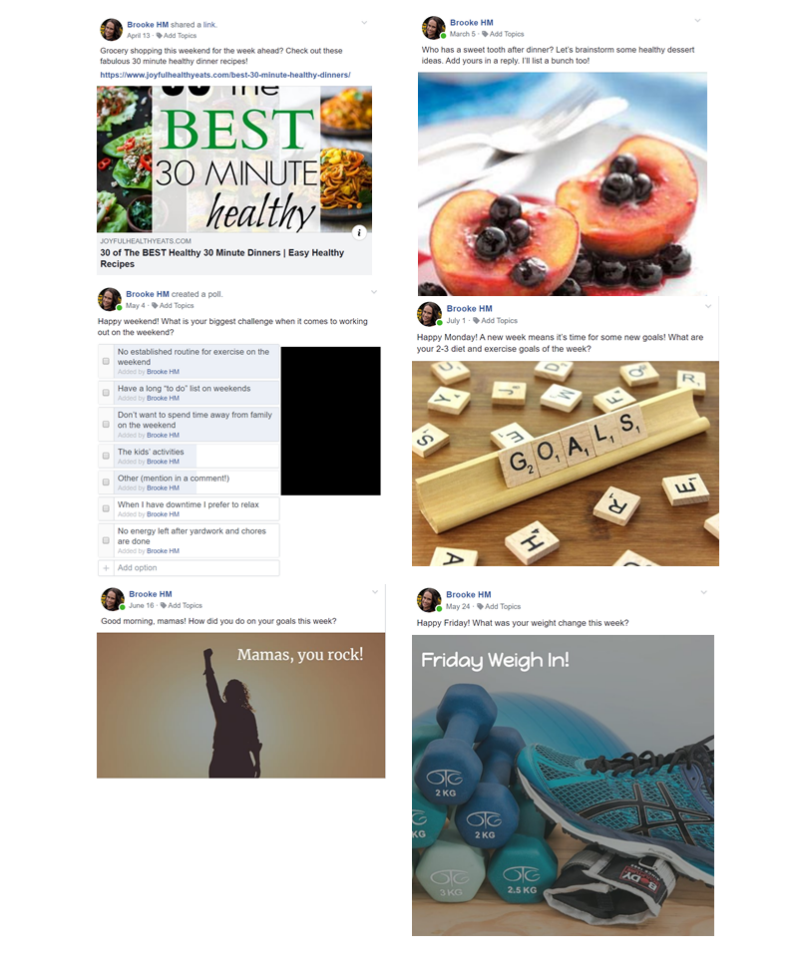
Sample intervention posts from the Facebook-delivered postpartum weight loss intervention by post type. For the middle post on the left, participants’ responses have been obscured with a black rectangle to protect participants’ confidentiality.

We will schedule daily intervention posts to be posted from the weight loss counselor’s account using the Facebook post scheduling tool. There will be 2 posts daily for the first 15 weeks, and 1 post daily for weeks 16 to 25, corresponding to the decrease in contact in the in-person condition. Intervention posts have been developed based on our previous research [30,65-67] to cover the intervention content in that intervention module of the DPP lifestyle intervention through posts that provide information or resources, solicit sharing of thoughts or experiences or challenges related to the topic of the week, ask participants to set goals (posted on Mondays), ask participants to report their progress toward these goals (posted on Sundays), and ask participants to report their weekly weight change (posted on Fridays). Sample intervention posts are shown in Figure 1. The weight loss counselor will facilitate discussions about weekly topics by engaging participants in problem solving, assisting them in setting SMART goals (ie, goals that are Specific, Measurable, Attainable, Relevant, Time-Based), providing constructive feedback, sharing resources, providing positive reinforcement, and answering questions.

### Assessments

### Weekly Surveys

Weekly during the intervention, participants in both treatment conditions will complete a brief (5-min) online survey to report current weight; app-tracked time on Facebook over the past 7 days; and their Facebook use habits, including what device(s) they used to access Facebook, what proportion of use was from their smartphone (vs other devices), and the extent to which other people used Facebook on their phone [46].

#### Six-Month Follow-Up Assessment

At 6 months, immediately following the intervention, participants will attend a focus group with other members of their weight loss group. Research staff will measure participants’ weight at the focus group visit. We will ask participants in both treatment conditions to elaborate on barriers and facilitators to participation. Participants in the Facebook condition will be asked for their reactions to and ratings of intervention posts that received low engagement. We will use their feedback to refine the format of low-engagement intervention posts to make them more engaging. Participants who do not attend the focus group will complete an individual follow-up visit that includes measurement of weight and an individual interview.

At 6 months, participants will also complete an online survey that includes assessment of current pregnancy, depressive symptoms [34], quality of life [40,41], infant feeding [42,43], social support for diet and physical activity changes [44], social media use [45], group cohesion [68,69], contamination, and intervention acceptability [30]. Participants will also report app-tracked time on Facebook over the past 7 days and their Facebook use habits, including what device(s) they used to access Facebook, what proportion of use was from their smartphone (vs other devices), the extent to which other people used Facebook on their phone [46], and whether they consciously changed their Facebook use as a result of becoming aware of the time spent on Facebook and, if so, in what way [70].

We expect that this follow-up visit will take 1 to 1.5 hours, and the survey will take 30 to 45 min. We will email participants a US $40 gift card after they complete the focus group and survey.

#### Twelve-Month Follow-Up Assessment

At 12 months (6 months after the end of the intervention), participants will attend a follow-up visit to measure weight and complete an online survey. Research staff will provide participants with instructions on how to remove the MyFitnessPal and time tracking apps from their phones. We will email participants a link to complete an online survey that includes measures of current pregnancy, depressive symptoms [34], quality of life [40,41], infant feeding [42,43], social support for diet and physical activity changes [44], and social media use [30,45]. Participants will also report app-tracked time on Facebook over the past 7 days and their Facebook use habits, including what device(s) they used to access Facebook, what proportion of use was from their smartphone (vs other devices), and the extent to which other people used Facebook on their phone [46]. We expect that the survey will take about 30 min, and the visit will take about 15 to 45 min. We will email participants a US $40 gift card after they complete the study visit and survey.

### Measures

#### Primary Outcomes: Feasibility

We will examine the feasibility of recruitment, sustained participation, contamination, retention, and feasibility of assessment procedures, particularly measurement of costs related to delivering and receiving the intervention.

#### Recruitment

We will calculate recruitment rates from the number of individuals contacted, screened, consented, and randomized, overall and by recruitment source. We will record the reasons for ineligibility and nonparticipation, including unwillingness to be randomized to either an online or in-person condition.

#### Sustained Participation

We will assess sustained participation in the intervention, that is, treatment retention. For the in-person condition, we will calculate sustained participation as time to last intervention session attended. Attendance at in-person intervention sessions will be recorded by the weight loss counselor at each meeting. For the Facebook condition, we will calculate sustained participation as time to last post, comment, or reaction (based on the date of post or comment reacted to) in the Facebook group. We will download objective engagement data from Facebook and calculate the date of last engagement.

#### Contamination

At 6 months, participants will complete a survey that includes questions about whether they participated in other weight loss programs (online or in-person) and whether they sought weight loss support on Facebook or other online social networks [71]; and, if so, to what extent and reasons why they sought this support.

#### Retention

We will calculate retention as the proportion of participants who complete the 6- and 12-month follow-up assessments in each condition.

#### Feasibility of Assessment Procedures

We will examine the extent and mechanisms of missing data on each measure to be included in the subsequent noninferiority trial, especially procedures used to capture costs. We will systematically track costs associated with delivery of both intervention conditions, capturing information on the costs that would be required to implement each intervention in practice (ie, outside the research context) using methods developed by others [72-74]. We have created an accounting system that captures administrative (eg, setting up the Facebook group and copying participants’ handouts), intervention delivery (eg, leading in-person intervention meetings and counseling via the Facebook group), and participant costs (eg, travel time to intervention meetings, time spent attending intervention meetings, and time spent on Facebook to participate in the intervention) for both conditions [75]. We will document any challenges with procedures for collecting data, including study records, participant-reported measures, measurement of weight, engagement data from Facebook, and time data recorded from tracking tools.

### Exploratory Outcome: Weight Change

At baseline, 6 months, and 12 months, weight will be measured twice and averaged. We will calculate percent weight change from baseline to 6 months and from baseline to 12 months. We will define clinically significant weight loss as 5% or greater [76,77] and will calculate the proportion of participants achieving this degree of weight loss at both follow-up points. For women who become pregnant during the study, we will use self-reported prepregnancy weight as their follow-up weight.

### Treatment Fidelity and Participant Safety

#### Treatment Fidelity

We will randomly select 20% of intervention modules, stratified by phase of the intervention: in-person group meetings weekly (weeks 1-15) or every other week (weeks 16-25). Study staff will review audiotapes from the in-person group sessions and content library of intervention posts from the Facebook condition and rate whether content objectives were met in each module. The weight loss counselor will receive feedback for intervention modules with less than 90% adherence to the protocol.

#### Participant Safety

The possible risks of participating in this study include an injury while being physically active, possible discomfort from completing questionnaires, and breach of confidentiality. Participants will be screened for ability to engage in physical activity, and we will obtain medical clearance from each participant’s primary care provider or obstetrician/gynecologist. Participants will be encouraged to participate in physical activity they are comfortable with, while gradually scaling to meet intensity recommendations, and avoid any exercises that could lead to injury, pain, or discomfort. Participants who experience injury or discomfort from exercise will be asked to meet with a health care provider before returning to physical activity*.* We will keep study data stored on REDCap [36], on password-protected research drives, or in locked filing cabinets. We will encourage participants to review privacy policies of Facebook, MyFitnessPal, and any time tracking apps, and during the informed consent process, we will review with participants the privacy considerations of participating in an intervention delivered via a secret Facebook group (eg, who can see what they post and what access Facebook has to content they share in the group). We will record and follow up on any adverse events that occur during the intervention, regardless of likely relation to the intervention. We will report any events that are related or possibly related to study procedures to the University of Connecticut IRB within 24 to 48 hours regardless of level of severity, and all other adverse events those that are not related or unlikely related to study procedures, during annual continuing IRB review.

Although not expected to be related to study participation, over the course of the study, research staff may become aware of elevated depressive symptoms among our participants, as depression is common among postpartum women [78]. Study assessments at baseline, 6 months, and 12 months include the EPDS [34]. Elevated depressive symptoms and/or suicidal ideation at baseline are exclusionary. The weight loss counselor will monitor participants’ posts on the Facebook group and discussions at in-person intervention sessions for disclosures of feelings of depressed mood during the intervention phase and will call participants disclosing such feelings to assess for clinical depression and make appropriate referrals if necessary. At any point during the study, we will refer participants for mental health care and/or arrange immediate psychiatric evaluation, as appropriate, and will alert referring providers of elevated depressive symptomology as identified by the EPDS [34].

### Statistical Analysis

We will use REDCap [36] to administer participant surveys and for participant tracking and data management. We will use SAS 9.4 (SAS Institute, Inc) to analyze quantitative data. Reporting of the feasibility outcomes and exploratory outcome of weight change will be descriptive (eg, percent retention at 6 and 12 months).

### Primary Outcomes: Feasibility

Examination of feasibility outcomes will inform the design of the subsequent full-scale noninferiority trial.

#### Recruitment

We will report recruitment using a Consolidated Standards of Reporting Trials (CONSORT) diagram [79,80]. We will compare yield from different recruitment approaches and will examine whether any eligibility criteria are excluding an inordinate proportion of otherwise eligible women. In particular, we will examine the number of otherwise eligible women excluded from participation because one or the other delivery mode is not feasible or unwillingness to be randomized to either treatment condition. If recruitment rates are lower than expected, we will adjust recruitment timelines as we plan the subsequent noninferiority trial.

#### Sustained Participation

We will compare sustained participation in both conditions. We will modify the delivery of both intervention conditions to address logistical and other barriers to sustained participation.

#### Contamination

We will describe the extent of contamination and reasons women sought these extra sources of support. If a significant proportion of women report seeking weight loss support from other sources, we will adapt our intervention to better meet their needs to reduce the occurrence of contamination in the subsequent trial.

#### Retention

We will report retention rates in each condition using a CONSORT diagram [79,80]. If retention is lower than expected, we will explore reasons for dropout and make changes to the protocol to address these challenges. We will also examine the proportion of participants who become pregnant during the study period.

#### Feasibility of Assessment Procedures

We will examine the extent and mechanisms of missing data, particularly measures of costs associated with delivering and receiving the intervention. For measures with an unacceptable amount of missingness, we will consider alternative measures in the subsequent noninferiority trial. We will resolve any issues arising in the collection of cost-related data and develop procedures for assessing time spent on Facebook to participate in the intervention.

#### Weight Loss (Exploratory)

We will report average percent weight loss in each treatment condition and the proportion that achieved clinically significant weight loss (≥5%).

#### Power

The purpose of this pilot trial is to examine the feasibility, thus identifying modifications required before examining noninferiority in a large randomized controlled trial. Leon et al [81] state that “power analyses should not be presented in an application for a pilot study that does not propose inferential results.” As they and others recommend [81,82], we based the sample size on necessities for examining the feasibility, thus identifying modifications required to the design of the trial or study procedures before conducting a full-scale noninferiority trial. Conducting 2 waves allows us to iteratively refine how we deliver intervention content via Facebook while assessing the feasibility of recruitment and engagement under the conditions of the subsequent trial. Although we will aim to maximize retention in both conditions, with retention of 80% or greater to be acceptable, a priori, we decided that a retention rate in either condition lower than 60% would indicate that the noninferiority trial is not feasible as designed. With 36 participants per treatment condition, the 95% CI for the estimated retention rate will be within ±13% if observed retention is 80%. Given 36 participants per condition, the lower limit of the 95% CI for the observed retention rate in either treatment condition should not be lower than 60%.

Owing to the variability in estimated standard errors from pilot studies, estimated effect sizes from small pilot studies should not be used to determine sample size to adequately power randomized controlled trials to assess intervention efficacy [81,83]. Thus, as recommended [81,83], we will calculate sample size requirements for the subsequent full-scale noninferiority trial based on a clinically meaningful noninferiority margin for percent weight loss and variance in weight loss observed in previous adequately powered trials of postpartum weight loss interventions [84].

## Results

Recruitment began in September 2018. The first wave of the intervention began in February 2019, and the second wave of the intervention began in October 2019. We anticipate completing follow-up assessments in fall 2020, and results will be analyzed at that time.

## Discussion

This randomized feasibility pilot trial will provide the necessary data to support a fully powered noninferiority trial comparing the Facebook-delivered postpartum weight loss intervention with traditional in-person delivery. A feasibility trial is important for several reasons. First, we need to demonstrate the feasibility of recruiting a sample of postpartum women who are willing and able to participate in either treatment condition. Barriers to attending numerous in-person sessions include work schedules, lack of childcare, and unreliable transportation [14-20], which may limit the available participant pool. In addition, women who have a strong preference for 1 condition may be more likely to drop out of treatment if they do not get randomized to their preferred condition. For this reason, we need to test recruitment and enrollment procedures to ensure adequate pacing of recruitment of eligible postpartum women who are able and willing to participate in either the in-person or Facebook condition to achieve the higher sample size required for efficacy testing.

Second, we need to establish that we can sustain participation in the Facebook-delivered condition adequately for 6 months. Previous studies using social media platforms for weight loss intervention delivery were either shorter than 6 months or had highly variable engagement [85]. In our 1-arm pilot of a 12-week version of the Facebook-delivered intervention, 63% of women sustained participation until the last week of the intervention, including 42% who engaged on the last day of the intervention [30]. Although this rate of treatment retention is promising, this study will provide information about sustained participation in the intervention throughout the full 6-month intervention. We have refined our intervention content after pretesting it in this and other pilot studies by identifying posts that received low engagement and rewording them to be more succinct; adding high-quality graphics; and including features known to elicit engagement such as polls, gifs, and challenges [65,86]. As attrition from treatment is a common challenge in postpartum weight loss intervention studies regardless of treatment modality [12,13], this study will also provide an opportunity to examine sustained participation in the in-person condition.

Third, we need to establish the feasibility of assessment procedures, particularly the measurement of costs related to delivering and receiving the intervention. We will systematically track costs that would be required to implement each intervention in practice (ie, outside the research context) [72-74] using a tracking system and procedures we previously developed [67]. The time participants spend using Facebook to participate in the intervention is a critical component of estimating the cost of receiving the intervention. Many Facebook users log in multiple times a day for short periods to scan their feed [87]. Within those periods, only a fraction of time might be dedicated to reading intervention posts and engaging with the counselor and other participants. Currently, no best practice exists for accurately measuring time spent on a particular Facebook feed [88]. We propose to use a difference-in-differences approach to compare changes in total Facebook use from preintervention across treatment conditions to estimate time spent to participate in the Facebook-delivered intervention. This approach requires accurate measures of total time on Facebook throughout the intervention for participants in both treatment conditions. As part of this project, we will develop procedures for measuring time spent on Facebook to participate in the intervention that maximize accuracy while not placing undue burden on participants nor changing their behavior as a result of surveilling their social media activities [70]. We will ask participants in both conditions to report the average time spent using Facebook recorded by the Facebook app’s time tracking tool or another app for tracking app usage and compare changes in Facebook use between the conditions over the course of the study. We will also query use patterns (eg, from other devices) and use this information to explore the likely accuracy of app-tracked time on Facebook. This formative work is needed before moving to a large-scale trial to assess cost-effectiveness.

Both social media and in-person treatment modalities have advantages and disadvantages that may impact efficacy for weight loss. The Facebook-delivered condition, by not requiring group visits, has the advantage of allowing participants to receive intervention content and participate in discussions where and when it works for them and providing more ready access to support from the counselor and other moms. They also do not need to carve out time in their schedule to attend intervention sessions, which may result in more time available for lifestyle activities such as exercise and meal preparation. A disadvantage is that intervention content competes with other highly engaging content in participants’ Facebook feeds and can easily be scrolled past. To compete for attention, intervention posts should be designed using similar features as other content on social media (eg, high-quality images). To this end, our team applies popular social media marketing trends when designing posts, and solicited feedback from postpartum women on intervention posts that received low engagement in our previous pilot study to refine intervention posts to elicit greater engagement from participants. Although an advantage of using a commercial social media platform for intervention delivery is the ability to capitalize on Facebook’s attractive technology (website and mobile app) and software for scheduling content, intervention research using Facebook as a delivery platform can be complicated by Facebook’s proprietary algorithm that determines how content is prioritized. Recently, Facebook announced an effort to put greater priority on content from Facebook groups; however, researchers have no way of knowing how much content ends up in each participants’ newsfeeds and have no ability to control that. New post notifications can be set up to circumvent this challenge. To the extent that data support the efficacy of Facebook-delivered intervention approaches, privacy protection will need to be secured before delivery in clinical settings.

An advantage of in-person delivery is that participants who attend receive face time with the counselor and each other, which provides more opportunity to develop tighter interpersonal bonds. Emotional support can be expressed through nonverbal behaviors such as smiling, nodding, and eye contact, all of which cannot be communicated online. Social media platforms attempt to fill this gap by allowing users to use clickable reactions (eg, like button) and gifs that create emotion through 1- to 2-second animations. A disadvantage is that missed visits are fairly common and, when repeated, often lead to attrition from treatment. This negatively affects treatment receipt and ultimately outcomes. Barriers to attending in-person sessions include work schedules, lack of childcare, and unreliable transportation [14-20]. Given the unique advantages and disadvantages of each treatment modality, we hypothesize that delivery via Facebook will not be appreciably worse on weight loss outcomes compared with delivery via in-person groups.

We hypothesize that social media delivery will be more cost-effective (ie, cost per participant, per kg lost, and per participant who lost ≥5%) than in-person delivery. Online interventions do not require physical space or travel time for interventionists or participants. In an era of limited health care dollars, data on cost-effectiveness can influence decision making about the provision of treatments or preventive interventions and the willingness to pay for these programs [73,74]. Previous cost-effectiveness analyses of weight loss interventions have suggested that technology-based approaches may be cost-effective compared with traditional in-person counseling [89,90], but available data on technology-delivered weight loss interventions are sparse [91]. This feasibility pilot trial will provide information on the feasibility of collection of cost-related data needed to evaluate cost-effectiveness of the Facebook-delivered intervention compared with in-person delivery in a subsequent large-scale noninferiority trial. Demonstration of cost-effectiveness in addition to efficacy can support the coverage of online lifestyle interventions by employers and health insurers.

### Conclusions

In summary, results from this randomized feasibility pilot trial will inform the design of a large-scale randomized controlled trial to assess whether delivering a postpartum weight loss intervention via Facebook is noninferior for weight loss and more cost-effective than delivering the intervention via traditional in-person groups. Following the noninferiority trial, we will assess the effectiveness of the Facebook-delivered intervention when implemented in real-world settings. Efficacious, cost-effective, and scalable strategies for postpartum weight loss have potential for high impact on the obesity epidemic and long-term maternal health.

## Appendix

Multimedia Appendix 1 Peer-review report from NIH for Grant R34HL136979.

## Abbreviations

BED: binge eating disorder
CONSORT: Consolidated Standards of Reporting Trials
DPP: Diabetes Prevention Program
EPDS: Edinburgh Postnatal Depression Scale
NIH: National Institutes of Health
IRB: institutional review board
PI: principal investigator
REDCap: Research Electronic Data Capture
