# Supplementary material for: Delivering a Post-Partum Weight Loss Intervention via Facebook or In-Person Groups: Protocol for a Randomized Feasibility Pilot Trial
Source: JMIR Res Protoc. 2019 Nov 28;8(11):e15530. doi: 10.2196/15530 (PMC6908979; doi:10.2196/15530)
Supplement: Multimedia Appendix 1 [file resprot_v8i11e15530_app1.pdf]

SUMMARY STATEMENT
(Privileged Communication)

PROGRAM CONTACT:
Sonia ARTEAGA
301-435-6677
arteagass@mail.nih.gov

Release Date: **11/18/2016**
Revised Date:

Application Number: **1 R34 HL136979-01**

Principal Investigator

WARING, MOLLY E.

Applicant Organization: UNIV OF MASSACHUSETTS MED SCH WORCESTER

Review Group: **ZHL1 CSR-G (F1)**
National Heart, Lung, and Blood Institute Special Emphasis Panel
NHLBI Clinical Trial Pilot Studies (R34)

Meeting Date: **10/27/2016**
Council: **JAN 2017**
Requested Start: **04/01/2017**
RFA/PA: **PAR16-037**
PCC: **HHCP N**

Project Title: **Delivering a Post-Partum Weight Loss Intervention via Facebook vs In-Person Groups: a Feasibility Pilot Trial**
SRG Action: **Impact Score:30**
Next Steps: **Visit http://grants.nih.gov/grants/next_steps.htm**
Human Subjects: **30-Human subjects involved - Certified, no SRG concerns**
Animal Subjects: **10-No live vertebrate animals involved for competing appl.**
Gender: **2A-Only women, scientifically acceptable**
Minority: **1A-Minorities and non-minorities, scientifically acceptable**
Children: **1A-Both Children and Adults, scientifically acceptable**
Clinical Research - not NIH-defined Phase III Trial

Project Year	Direct Costs Requested	Estimated Total Cost
1	150,000	251,250
2	150,000	251,250
3	150,000	251,250
TOTAL	450,000	753,750

ADMINISTRATIVE BUDGET NOTE: The budget shown is the requested budget and has not been adjusted to reflect any recommendations made by reviewers. If an award is planned, the costs will be calculated by Institute grants management staff based on the recommendations outlined below in the COMMITTEE BUDGET RECOMMENDATIONS section.

1R34HL136979-01 WARING, MOLLY

RESUME AND SUMMARY OF DISCUSSION: An excellent pilot clinical trial application proposes to plan to refine an existing intervention and assess the feasibility of conducting a 2 arm randomized controlled trial that will administer a Facebook delivered weight management intervention and an in person version of weight management intervention among postpartum women. The committee discussion noted the significance of post-partum weight loss and the utility of comparing a weight loss intervention using a low cost social media approach with the standard in-person approach. While there was concern regarding the use of a non-inferiority design, the inclusion of the cost effectiveness component added to the potential significance of the results. The investigative team is excellent and very capable while the environment appears very supportive. The innovation of using Facebook as the platform was good, however it was noted that this platform lacked extensive ability to monitor use while the lack of potential generalizability to other platforms was also of concern. The approaches to be taken also appeared to raise some concerns including how the proposed results would inform and lead to a future clinical trial in this specific patient population especially relative to the selection and training and monitoring of the intervention leaders, the non-specific nature of the outcomes as well as responding to participant depression issues. Overall the review panel viewed the application as having potentially high impact level.

DESCRIPTION (provided by applicant): Post-partum weight retention contributes to obesity for many women, increasing risk for cardiovascular disease and other chronic diseases and complicating future pregnancies. Lifestyle interventions have shown to be modestly efficacious for post-partum weight loss in randomized controlled trials, yet interventions with numerous visits are logistically challenging for many post-partum women. Innovative and efficacious treatment models for post-partum weight loss that fit into the busy lives of new moms are needed, and cost-effectiveness is critical for adoption. Facebook may be an efficient platform for delivering evidence-based weight loss programming to post-partum women. Delivering interventions via Facebook allows us to connect with post- partum women where they are, more fully integrating into their lives and daily routines. We have developed a post-partum weight loss intervention based on the Diabetes Prevention Program, tailored to needs of post-partum women and for delivery via Facebook. The goal of the proposed project is to gather critical preliminary data to finalize the design of a large randomized trial to compare the non-inferiority and cost-effectiveness of this post-partum weight loss intervention delivered via Facebook versus in-person group sessions. We will conduct a pilot randomized trial with 72 overweight or obese post-partum women comparing delivery of a post- partum weight loss intervention via Facebook to in-person group sessions. We will examine the feasibility of recruitment (especially the proportion of women unwilling to be randomized to one condition), sustained participation, contamination, retention, and feasibility of assessment procedures, particularly measurement of cost-related data, in both treatment conditions. We will describe weight loss as an exploratory outcome. Second, in pre-pilot and post-intervention focus groups, we will solicit women's feedback on posts with low engagement, and will iteratively refine these posts to make them more engaging. Finally, we will compare self- reported time spent on Facebook to participate in the intervention with application-tracked time on Facebook, changes in use, and time spent visibly engaging, to develop procedures for measuring time spent on Facebook to participate in the intervention that balance accuracy and participant burden. The proposed project will provide preliminary data needed to finalize the design of a subsequent large non-inferiority trial. Demonstrating cost-effectiveness in addition to efficacy of our Facebook-delivered post-partum weight loss intervention is critical to support widespread implementation. **PUBLIC HEALTH RELEVANCE:** The goal of the proposed work is to test the feasibility of a post-partum weight loss intervention delivered via Facebook or in-person group sessions. We will also iteratively refine the Facebook intervention to increase participant engagement and develop procedures for measuring time spent on Facebook to participate in the intervention that balance accuracy and participant burden. The proposed project will provide critical preliminary data to finalize the design of a large randomized trial to compare the non-inferiority and cost-effectiveness of our Facebook-delivered post-partum weight loss

intervention. If efficacious and cost-effective, our Facebook- delivered intervention leads naturally to strategies for scaling up for widespread impact. (End of Abstract)

CRITIQUE 1:

Significance: 2
Investigator(s): 1
Innovation: 3
Approach: 2
Environment: 1

Overall Impact: The applicants have chosen an important area for study. One concern is that they propose a non-inferiority trial to be compared to an approach that is described as only modestly effective. This is especially important as many women enter pregnancy obese and ultimately need more substantial postpartum weight management. On the other hand, the proposed approach using Facebook may be less costly and more convenient for postpartum mothers.

The applicants have clearly identified information that is needed to design and complete the subsequent trial. One aspect that is not as well developed is the cost-effectiveness component. The investigative team and the environment appear to be well suited to the proposed research.

1. Significance:
Strengths
- Postpartum weight gain and maintenance of that weight gain has important ultimate health implications for women.

Weaknesses
- The proposed research must be viewed in the overall context of obesity in women of childbearing age. The proposed study will address only a relatively minor component of the overall problem.

2. Investigators:
Strengths
- The investigative team is outstanding with appropriate expertise for the proposed project.

Weaknesses
- None

3. Innovation:
Strengths
- The use of social media has some innovative aspects.

Weaknesses
- Overall, the project does not present particularly novel approaches.

4. Approach:
Strengths
- There are numerous strengths to the proposed approach. The applicants have already completed a single arm pilot study.
- The applicants have clearly outlined the elements needed to design and conduct the subsequent trial.

Weaknesses
- The elements needed to design and execute the cost-effectiveness aspect of the study are not well developed.

- More detail regarding how the proposed trial will inform the sample size for a non-inferiority study would be useful.

5. Environment:
Strength
- The environment for the proposed investigation appears to be outstanding.

Weaknesses
- None noted

CRITIQUE 2:

Significance: 3
Investigator(s): 2
Innovation: 3
Approach: 5
Environment: 2

Overall Impact: The investigators propose a pilot clinical trial to inform a subsequent non-inferiority trial comparing effectiveness and cost of a Facebook focused approach and an in-person visit approach to postpartum weight loss. The investigators will use a user-centered approach to inform the intervention refinement. However, a more detailed description of the intervention refinement process is needed to enhance the scientific rigor. Additionally, the pilot clinical trial has several methodological flaws. Of these methodological flaws, the potential of contamination, the assignment of counselors to both experimental conditions, selection bias, and several uncontrolled sources of measurement error draw concerns.

1. Significance:
Strengths
- The retention of postpartum weight can contribute to obesity and increased cardiometabolic risk among women.
- Traditional weight management programs are delivered in person. However, postpartum mothers are unlikely to attend in-person weight management programs consistently due to caregiving and other demands.
- This proposed clinical trial will attempt to use a social media platform that is widely accepted and actively used among postpartum mothers for social support.

Weaknesses
- The investigators admit that it is unlikely that a Facebook delivered intervention will have superior benefits to traditional weight management programs.
- It is unclear how the current proposed clinical trial or the subsequent confirmatory trial will advance this area of behavior change science. For instance, women can already access weight management programs online with formal and peer supports.

2. Investigator(s):
Strengths
- The PI, Dr. Waring, is an epidemiologist and assistant professor. She qualifies as a NIH New and Early Stage Investigator. She has experience with conducting pilot clinical trials in weight management and delivering interventions through social media platforms.
- This is a relatively established team of investigators. In particular, Drs. Simas-Moore, and Pagoto have a record of publication with Dr. Waring.
- Dr. Wang (consultant), assistant professor of economics, states that his contribution to the project is to monitor cost-related data collection and conduct cost-related data analyses.

Weaknesses

- The statistician on this project has relatively few publications and no recent funding.

3. Innovation:
Strengths
- The investigators aim to leverage the usage of an online social media platform, Facebook, to deliver a postpartum weight-loss intervention.
- If there are promising results from the proposed trial and larger confirmatory trial, there intervention has the potential for large population scalability.

Weaknesses
- Despite the adaption of an established Diabetes Prevention Program for postpartum women to attenuate weight gain and cardiometabolic risk, this proposed project does little to advance behavioral science focused on weight-loss.
- Although Facebook is a widely used social media platform, the investigators do not fully address the issue of capturing the 'dose' of their intervention or the participant's time spent viewing study specific Facebook posts. Instead of directly addressing the issue, the investigators aim to use a combination of self-report measurements and general measures of Facebook use to describe the 'dose' or exposure to the Facebook experimental condition. Thus, if the investigators could have directly overcome the issue of capturing the dose or time exposed to Facebook condition resources, then this study would provide a methodological innovation that could advance eHealth intervention research using social media platforms.

4. Approach:
Strengths
- The investigators provide sufficient justification of how the current R34 application will inform a subsequent clinical trial.
- A user-centered approach is specified to assist with the refinement of the intervention, which may offer some insights on how to enhance engagement among participants randomly assigned to the Facebook experimental condition.
- Although not explicitly stated, this project is designed as an unblinded two-arm randomized controlled trial and participants will be randomized using a permutated block design to controlled for variance in the numbers of postpartum days by participant.
- Statistical analysis plan is consistent with the specific aims and well-explained.

Weaknesses
- There are several methodological concerns that affect the scientific rigor of the proposed clinical trial. For instance, in specific aim 1, there is reported that cost-related data will be analyzed but there is no mention of what cost-data related data will be captured or even what specific analytic techniques will be performed.
- A lack of scientific rigor regarding the conduct the focus group(s). In particular, research strategy does not clearly outline the procedures for the analysis of the qualitative data, validation of the trustworthiness of these qualitative data, or provide a detailed description of the process that would be used among the investigators to inform their decision making. There is no justification for the sample size (N = 10) proposed for the intervention refinement focus groups.
- Similarly, there is no justification for the sample size of 72 participants for the pilot clinical trial.
- The investigators provide a sufficient review of the extant literature. Yet, the scientific premise of the intervention condition, its components and specification of dosage is not clearly articulated and mechanisms of how the interventions are expected to influence weight-loss are simply not presented.
- Given the incidence of postpartum depressive symptoms that may alter health promotion behaviors, the investigators fail to address the phenomenon. There is no discussion of the incidence of postpartum depressive symptoms and the potential confounding effects on weight loss or physical activity.

- It is unclear how the investigators will account for social determinants of health (e.g., race/ethnicity, insurance, employment status, education, broadband/wireless access, financial resources, household demands, and financial strain) will impact the feasibility of the project or representativeness of the qualitative results for the intervention refinement process. Analysis of subgroups would be insightful and relevant to specific aim 1.
- The investigators state that there is no difference in the content of the experimental condition. Yet, plan to use counselors to administer resources across study conditions. A stronger design would be to isolate counselors to an experimental condition to minimize a threat to the internal validity of this study.
- Given the threat of contamination, it is unclear why the investigator chose 6-months to evaluate if participants were exposed to other weight-loss programs.
- It is unclear what the investigators will do if engagement in either experimental condition is low. There are no benchmarks noted or procedures outlined on how to sustain engagement in either condition.
- The investigators provide no rationale for the selection of instruments or procedures to reduce measurement error of the exploratory outcome of weight and other variables (e.g., Facebook time, physical activity monitoring, dose exposure in the Facebook condition).
- Given the importance of the counselor, there is insufficient description of the counselor’s background (e.g., dietician, nurse, or paraprofessional), which can affect the delivery of the counseling on perceived emotional support and subject engagement. Additional information is needed about who this person is and time related costs.

5. Environment:
Strengths
- The investigative team has access to potential participants and the resources need to successful conduct this clinical trial.
- Based on the data provided, the investigators should have access to sufficient numbers of potential participants.

Weaknesses
- It is unclear what the demographic characteristics of women who receive care at the study site or the Worcester community. This may pose a significant threat to the external generalizability of the study.

CRITIQUE 3:

Significance: 2
Investigator(s): 1
Innovation: 1
Approach: 3
Environment: 1

Overall Impact: The aim of this interesting study is to pilot test a Facebook based version of the Diabetes Prevention Program (DPP) modified to address post-partum weight loss. This is a significant project as a substantial proportion of women gain weight during pregnancy and either fail to lose this weight or continue to gain weight in the early parenting years. Mothers’ weight status influences children’s obesity and mother’s health. Developing effective, easy-to-disseminate interventions to promote weight loss among new mothers has tremendous public health significance. Facebook is a social media platform used by a very high proportion of new parents, and could permits wide dissemination of the intervention should it prove successful. The intervention may be particularly useful for meeting the needs of busy and potentially overwhelmed new mothers. The PI has developed much of the protocol which is based on the DPP. The content of the intervention includes self-monitoring,

psychoeducation, links to resources to facilitate health promotion, use of activity trackers, and social support from group leaders and members of a private group created for participants on Facebook. The effects of the Face-book version of the intervention is compared to a face-to-face version of the same intervention delivered in a group format. Group formats have been demonstrated to support weight loss, the internet and Facebook are good sources of information and feedback.

A total of 10 participants will engage in focus group feedback discussions to facilitate modification of the program, and 72 participants will be assigned at random to either the Facebook or face to face interventions. Outcomes include measures of recruitment, feasibility, usage, and satisfaction, among other measures. Between-groups differences in weight loss will be assessed in an exploratory analysis. The PI is very strong and the investigative team has the expertise to engage in this project. They have biostatistics expertise, mHealth expertise, and health behavior change expertise, among other skills. They have been productive. A previous pilot study provided an opportunity to develop the protocols, but did not provide sufficient information to develop a full-fledged R01. The current study is necessary and generally would be sufficient to develop the R01. There is a good strategy for analyses and data management, and the PI has been very clear about the ways the information to be gathered in this trial will help develop the R01.

The primary concern is the lack of a plan for evaluating differential response to the two treatments. Some women may do better with FB interventions, others with in-person interventions. A pilot study cannot determine which interventions will be better for which women, but it can serve to identify possible theoretically or empirically-driven predictors. These predictors would enrich the value of the subsequent R01. The investigators have included some important variables that might predict differential response including social norms for weight loss and social support. But it would have been helpful to understand why they included these measures, and if they expect that availability of support predicts outcomes in response to one or the other interventions. Other variables may also be important predictors. For example, depression may serve as a barrier to the in person intervention but not the FB version. High levels of interpersonal sensitivity may serve as a barrier to the in person intervention but not the FB version. Distance from the clinic may also serve as a barrier to some types of participation, although ease of access and other neighborhood characteristics are not collected.

The investigators see contamination as a problem; however, obtaining weight loss support elsewhere could also be viewed as an index of patient activation and a positive sign.

There is very little information provided about the group leader or the interventions. No information is provided on the leaders qualifications or training. No information is provided on fidelity checks of intervention. This is critical since the leader will be conducting both types of interventions and may have opinions about which is better.

1. Significance:
Strengths
- Highly significant project as postpartum weight loss is difficult, and chronic obesity has long term health implications for the mother and her family.
- Facebook is a widely used social media platform, and research investigating its use as a public health tool is highly significant.
- This project is necessary to develop an R01. If this R01 demonstrates that a Facebook version of the Diabetes Prevention Program is an effective tool for weight loss in the postpartum period, it could change the nature of intervention delivery for new mothers. The FB version could be widely disseminated, permitting distance treatment.

Weaknesses
- There are limitations to the ability to detect predictors of differential response to the FB vs face-to face intervention.

- Without sufficient information about the group leaders, it is difficult to understand exactly how this would be implemented on a larger scale or sustained after the study is over.

2. Investigator(s):
Strengths
- Very strong team with an epidemiologist, psychologist, biostatistics, and expert in mHealth applications. There is good expertise for the interventions and for evaluating the cost effectiveness of the intervention.

Weaknesses
- None

3. Innovation:
Strengths
- This intervention is innovative. There is growing interest in the public health applications of Facebook. If these interventions are successful, they have the potential to permit widespread dissemination. The investigative team is planning to deploy a known intervention in a new media format this is an innovative approach. It will permit comparisons across different versions of this widely used program.

Weaknesses
- Others have used FB as a platform for intervention. However, it is still such a new approach to public health interventions that this is a minor issue.

4. Approach:
Strengths
- Overall a strong approach. The R34 will provide much of the information needed to develop and conduct an R01.
- The population is in need of intervention.
- The recruitment seems feasible. The resources for recruitment are strong
- The measures are good.
- The detailed evaluation of the knowledge to be gained is helpful.
- The plan for evaluating time spent on the interventions is thoughtful. The approach to assessing feasibility is good.
- The matching of the intervention between the FB version and the in-person version is excellent.
- The intervention components are very strong.
- The opportunity for referral to other resources is good.
- The use of “secret groups” is good.
- Assessing social norms for behavior change is excellent, even though the issues are not discussed in detail in the intervention. It will be very useful to understand if the groups created in FB and the groups created for the in person intervention have similar effects on social norms and perceived support.

Weaknesses
- There is very little information on the group leaders.
- It would be helpful to have assessments of variable which may predict differential response to the FB vs. in person intervention.
- It would probably be useful to know if women in the FB condition shared information about the intervention with friends and family (i.e., showed them the materials and asked them to go through the exercises together) more than the women in the in person condition.
- Contamination may have benefits and could be considered an index of patient activation.

5. Environment:
Strengths

- Very strong environment. There should be no problem recruiting participants.

Weaknesses

- None

CRITIQUE 4:

Significance: 2
Investigator(s): 2
Innovation: 1
Approach: 1
Environment: 1

Overall Impact: This is a well prepared comprehensive proposal addressing an important maternal health issue. Key issues are thoughtfully address and strategies based on previous research and experience. The study design is sound as are the implantation procedures including recruitment, retention, data collection and data analyses. Overall a very solid proposal with no major flaws.

1. Significance:
Strengths

- The proposal addresses an important and somewhat difficult issue of post-partum weight loss with a procedure that is possible less costly and less intrusive.

Weaknesses

- None

2. Investigator(s):
Strengths

- Although some members are somewhat junior, the full investigative team is strong, with appropriate experience for this pilot and fully capable of completing the proposed study.

Weaknesses

- None

3. Innovation:
Strengths

- The proposed use of Face Book as a strategy for intervention delivery is very innovative and, if successful, could lead to enhanced weight loss strategies.

Weaknesses

- None noted

4. Approach:
Strengths

- The overall approach is very strong, with a meaningful hypothesis and an appropriate design for providing the information required for a subsequent full scale trial.
- The statistical issues are very well addressed.
- The data management plans are very solid.
- Participant recruitment and retention plans are appropriate.
- The proposal carefully addresses the procedures for collecting the information needed for the design of the subsequent full scale trial.

Weaknesses

- None noted

5. Environment:

Strengths

- The host institution has all the resources and research environment required for the proposed study.

Weaknesses

- None noted

THE FOLLOWING SECTIONS WERE PREPARED BY THE SCIENTIFIC REVIEW OFFICER TO SUMMARIZE THE OUTCOME OF DISCUSSIONS OF THE REVIEW COMMITTEE, OR REVIEWERS' WRITTEN CRITIQUES, ON THE FOLLOWING ISSUES:

PROTECTION OF HUMAN SUBJECTS (Resume): ACCEPTABLE
Well prepared procedures and protections

DATA AND SAFETY MONITORING PLAN: ACCEPTABLE
Fully acceptable, includes the creation and use of a DSMB

INCLUSION OF WOMEN PLAN (Resume): ACCEPTABLE
Only women will be included.

INCLUSION OF MINORITIES PLAN (Resume): ACCEPTABLE
Minority and non-minority participants will be included.
A more detailed description of minority recruitment plan is needed. Included in this plan should be strategies (e.g., additional clinics, minority research staff, etc.) to achieve the racial/ethnic distribution of the Worcester county.

INCLUSION OF CHILDREN PLAN (Resume): ACCEPTABLE
Women age 18-21 will be included. Dietary requirements for younger mothers may be too different.

RESOURCE SHARING PLANS: UNACCEPTABLE
No plan is included.

COMMITTEE BUDGET RECOMMENDATIONS: The budget was recommended as requested.

Footnotes for 1 R34 HL136979-01; PI Name: Waring, Molly E.

NIH has modified its policy regarding the receipt of resubmissions (amended applications). See Guide Notice NOT-OD-14-074 at http://grants.nih.gov/grants/guide/notice-files/NOT-OD-14-074.html. The impact/priority score is calculated after discussion of an application by averaging the overall scores (1-9) given by all voting reviewers on the committee and multiplying by 10. The criterion scores are submitted prior to the meeting by the individual reviewers assigned to an application, and are not discussed specifically at the review meeting or calculated into the overall impact score. Some applications also receive a percentile

ranking. For details on the review process, see
http://grants.nih.gov/grants/peer_review_process.htm#scoring.

MEETING ROSTER
National Heart, Lung, and Blood Institute Special Emphasis Panel

NATIONAL HEART, LUNG, AND BLOOD INSTITUTE
NHLBI Clinical Trial Pilot Studies (R34)
ZHL1 CSR-G (F1)
10/27/2016

CHAIRPERSON(S)

ASHIKAGA, TAKAMARU, PHD
PROFESSOR AND DIRECTOR
DEPARTMENT OF MEDICAL BIOSTATISTICS
UNIVERSITY OF VERMONT
BURLINGTON, VT 05405

MEMBERS

BRONDOLO, ELIZABETH N., PHD
PROFESSOR
DEPARTMENT OF PSYCHOLOGY
ST. JOHN'S UNIVERSITY
JAMAICA, NY 11439

COUPER, DAVID J., PHD
CLINICAL PROFESSOR AND DEPUTY DIRECTOR
DEPARTMENT OF BIOSTATISTICS - CSCC
COLLABORATIVE STUDIES COORDINATING CENTER
UNIVERSITY OF NORTH CAROLINA AT CHAPEL HILL
CHAPEL HILL, NC 27514

DANIELS, STEPHEN R., MD, PHD
PROFESSOR AND CHAIRMAN
DEPARTMENT OF PEDIATRICS
CHILDREN'S HOSPITAL COLORADO
UNIVERSITY OF COLORADO
AURORA, CO 80045

DAVIGLUS, MARTHA L., MD, PHD
ASSOCIATE VICE CHANCELLOR FOR RESEARCH
DIRECTOR, INSTITUTE FOR MINORITY HEALTH RESEARCH
PROFESSOR OF MEDICINE
UNIVERSITY OF ILLINOIS
CHICAGO, IL 60612

FRAMPTON, MARK WALTER, MD
PROFESSOR EMERITUS
EMERITUS OF MEDICINE & ENVIRONMENTAL MEDICINE
PULMONARY & CRITICAL CARE
UNIVERSITY OF ROCHESTER MEDICAL CENTER
ROCHESTER, NY 14642-8692

HICKMAN, RONALD LEE JR, PHD
ASSOCIATE PROFESSOR
SCHOOL OF NURSING
CASE WESTERN UNIVERSITY
CLEVELAND, OH 44106

JACKSON, DANIEL J., MD
ASSOCIATE PROFESSOR OF PEDIATRICS
SECTION OF ALLERGY, IMMUNOLOGY & RHEUMATOLOGY
DEPARTMENT OF PEDIATRICS
UNIVERSITY OF WISCONSIN-MADISON
MADISON, WI 53792-9988

MILLER, J. PHILIP, AB
PROFESSOR
DIVISION OF BIOSTATISTICS
LEAD,RESEARCH DESIGN AND BIOSTATISTICS GROUP, ICTS
WASHINGTON UNIVERSITY SCHOOL OF MEDICINE
ST. LOUIS, MO 63110

MITCHELL, MICHAEL E., MD
PROFESSOR
MEDICAL COLLEGE OF WISCONSIN
9000 W. WISCONSIN AVENUE
MILWAUKEE, WI 53122

MOORE, CHRISTOPHER L., MD
ASSOCIATE PROFESSOR
DEPARTMENT OF EMERGENCY MEDICINE
YALE UNIVERSITY
SCHOOL OF MEDICINE
NEW HAVEN, CT 06520-8047

PAHL, ELFRIEDE (ELFI), MD
ME WODIKA RESEARCH PROFESSOR
NU FEINBERG SCHOOL OF MEDICINE
MEDICAL DIRECTOR HEART TRANSPLANTATION
LURIE CHILDRENS HOSPITAL
CHICAGO, IL 60611

REZNIK, MARINA, MD
ASSOCIATE PROFESSOR OF PEDIATRICS
THE CHILDREN'S HOSPITAL AT MONTEFIORE
MONTEFIORE MEDICAL CENTER
THE PEDIATRIC HOSPITAL
ALBERT EINSTEIN COLLEGE OF MEDICINE
BRONX, NY 10467

SIMS, MARIO, PHD
ASSOCIATE PROFESSOR OF MEDICINE
DEPARTMENT OF MEDICINE
JACKSON HEART STUDY
JACKSON MEDICAL MALL, STE 701
UNIVERSITY OF MISSISSIPPI MEDICAL CENTER
JACKSON, MS 39213

SOLTZ, BARBARA A., PHD
OWNER AND CEO
CONVERSION ENERGY ENTERPRISES (CEE)
SPRING VALLEY, NY 10977

SYMONS, HEATHER J., MD
ASSISTANT PROFESSOR
DEPARTMENT OF ONCOLOGY AND PEDIATRICS
PEDIATRIC BLOOD AND MARROW TRANSPLANTATION
CANCER RESEARCH BUILDING I
JOHNS HOPKINS UNIVERSITY
BALTIMORE, MD 21287-0013

VELAZQUEZ, ERIC J., MD
PROFESSOR
DIVISION OF CARDIOVASCULAR MEDICINE
DIRECTOR, CARDIAC DIAGNOSTIC UNIT
AND ECHOCARDIOGRAPHY LABOATORIES
DUKE CLINICAL RESEARCH INSTITUTE, DUKE UNIVERSITY
DUKE CLINICAL RESEARCH INSTITUTE, DUKE UNIVERSITY
DURHAM, NC 27705

WILLIAMS, OREN (O.) DALE, PHD
DEPARTMENT CHAIR
DEPARTMENT OF BIOSTATISTICS
ROBERT STEMPEL COLLEGE OF PUBLIC HEALTH
AND SOCIAL WORK
FLORIDA INTERNATIONAL UNIVERSITY
MIAMI, FL 33199

WU, ANN CHEN, MD
ASSISTANT PROFESSOR
OFFICE OF RESEARCH ADMINISTRATION
DEPARTMENT OF POPULATION MEDICINE
BOSTON, MA 02215

MAIL REVIEWER(S)

BROWN, ANNE WHITNEY, PHD
DIRECTOR
CYSTIC FIBROSIS AND BRONCHIECTASIS PROGRAM
INOVA ADVANCED LUNG DISEASE AND TRANSPLANT PROGRAM
INOVA HEALTH SYSTEM
FALLS CHURCH, VA 22046

SCIENTIFIC REVIEW OFFICER

KIM, CHANG SOOK, PHD
SCIENTIFIC REVIEW OFFICER
OFFICE OF SCIENTIFIC REVIEW/DERA
NATIONAL HEART, LUNG, AND BLOOD INSTITUTE
BETHESDA, MD 20892

EXTRAMURAL SUPPORT ASSISTANT

MCCORKLE, ATHENA M
EXTRAMURAL SUPPORT ASSISTANT
OFFICE OF SCIENTIFIC REVIEW/DERA
NATIONAL HEART, LUNG, AND BLOOD INSTITUTE
NATIONAL INSTITUTE OF HEALTH
BETHESDA, MD 20892

MCROY, CASSANDRA F.
EXTRAMURAL SUPPORT ASSISTANT
OFFICE OF SCIENTIFIC REVIEW/DERA
NATIONAL HEART, LUNG, AND BLOOD INSTITUTE
NATIONAL INSTITUTES OF HEALTH
BETHESDA, MD 20892

PROGRAM REPRESENTATIVE

ARTEAGA, SONIA S., PHD
PROGRAM DIRECTOR
DIVISION OF CARDIOVASCULAR SCIENCES
NATIONAL HEART, LUNG AND BLOOD INSTITUTE
NATIONAL INSTITUTE OF HEALTH
BETHESDA, MD 20892

BURNS, KRISTIN, BA
PROGRAM OFFICER
NHLBI/DCVS/PAPCR/JDSDB
NATIONAL INSITUTE OF HEALTH
BETHESDA, MD 20892

DESVIGNE-NICKENS, PATRICE, MD, PHD
MEDICAL OFFICIER
DIVISION OF CARDIOVASCULAR SCIENCES
NATIONAL HEART, LUNG, AND BLOOD INSTITUTE
NATIONAL INSTITUTES OF HEALTH
BETHESDA, MD 20892

DI FRONZO, NANCY L., PHD
PROGRAM DIRECTOR
BLOOD RESOURCES PROGRAM, DBDR
NATIONAL HEART, LUNG AND BLOOD INSTITUTE, NIH
NATIONAL INSTITUTES OF HEALTH
BETHESDA, MD 20892

EINHORN, PAULA, MD
MEDICAL OFFICER/PROGRAM DIRECTOR
DIVISION OF CARDIOVASCULAR SCIENCES
NATIONAL HEART, LUNG, AND BLOOD INSTITUTE
NATIONAL INSTITUTES OF HEALTH
BETHESDA, MD 20792

FREEMER, MICHELLE M., MD
PROGRAM REPRESENTATIVE
DIVISION OF LUNG DISEASES
NATIONAL HEART, LUNG AND BLOOD INSTITUTE
NATIONAL INSTITUTES OF HEALTH
BETHESDA, MD 20892

HARABIN, ANDREA L., PHD
SENIOR SCIENTIFIC ADVISOR
DIVISION OF LUNG DISEASES
NATIONAL HEART, LUNG, AND BLOOD INSTITUTE
NATIONAL INSTITUTES OF HEALTH
BETHESDA, MD 20892

KINDZELSKI, ANDREI L., PHD
PROGRAM OFFICER
TRANSLATIONAL BLOOD SCIENCE AND RESOURCES
BRANCH
DIVISION OF BLOOD DISEASES AND RESOURCES
NATIONAL HEART, LUNG, AND BLOOD INSTITUTE
BETHESDA, MD 20892

LATHROP, DAVID A., PHD
BRANCH CHIEF, HEART FAILURE & ARRHYTHMIAS
DIVISION OF CARDIOVASCULAR DISEASES
NATIONAL HEART, LUNG, AND BLOOD INSTITUTE
NATIONAL INSTITUTES OF HEALTH
BETHESDA, MD 20892

MACGARVEY, NANCY, MD
PROGRAM OFFICER
NATIONAL HEART, LUNG, AND BLOOD INSTITUTE
NATIONAL INSTITUTE OF HEALTH
BETHESDA, MD 20892

ROSENBERG, ELLEN, PHD
CLINICAL TRIALS SPECIALIST
DIVISON OF CARDIOVASCULAR SCIENCES
NATIONAL HEART, LUNG, AND BLOOD INSTITUTE
NATIONAL INSTITUTES OF HEALTH
BETHESDA, MD 20892

SOPKO, GEORGE, MD, MPH
MEDICAL OFFICER
DIVISION OF HEART AND VASCULAR DISEASES
NATIONAL HEART, LUNG, AND BLOOD INSTITUTE
BETHESDA, MD 20892

WEINMANN, GAIL, MD
DEPUTY DIRECTOR
DIVISION OF LUNG DISEASES
NATIONAL HEART, LUNG, AND BLOOD INSTITUTE
NATIONAL INSTITUTES OF HEALTH
BETHESDA, MD 20814

XIAO, LEI, MD
PROGRAM REPRESENTTIVE
NHLBI/DLD/LBDB
NATIONAL INSTITUTE OF HEALTH
NATIONAL HEART LUNG AND BLOOD INSTITUTE
BETHESDA, MD 20892

Consultants are required to absent themselves from the room during the review of any application if their presence would constitute or appear to constitute a conflict of interest.
